# Physician self-identified race and opioid prescription practices in upper extremity injuries in the pediatric emergency department

**DOI:** 10.1016/j.heliyon.2023.e13351

**Published:** 2023-02-02

**Authors:** Joseph Langham, Sherita Holmes, Janet Figueroa, Srikant Iyer, Sarah Lazarus, Scott Gillespie, Carmen Sulton

**Affiliations:** a Emory University School of Medicine, Pediatric Emergency Medicine, Atlanta, GA, USA; b Emory University School of Medicine, Atlanta, GA, USA; c Children's Healthcare of Atlanta, Emory University School of Medicine, Atlanta, GA, USA; d Pediatric Emergency Medicine Physician, Atlanta, GA, USA

**Keywords:** Pediatrics, Emergency medicine, Pediatric emergency medicine, Analgesia, Pain management, Long bone fractures, Procedural sedation, Disparities

## Abstract

**Background:**

Minority children have been shown to receive fewer opioid analgesics for acute pain.

**Objective:**

Assess if both White and non-White physicians prescribe fewer opioids to non-White children presenting to the pediatric emergency department (PED) with upper extremity (UE) fractures.

**Methods:**

Patients with acute UE fractures were evaluated. Attending physicians provided their self-identified race and consented to analysis of their opioid prescribing practices. Primary outcome was receipt of an opioid prescription at discharge. Bivariate analyses measured the association between patient race and receipt of an opioid prescription; further analysis evaluated the effect of physician race on prescription practices. Generalized linear models measured these associations while controlling for confounders.

**Results:**

Thirty-four percent of eligible patients (2754/8155) were discharged with an opioid prescription. There was no statistically significant difference in odds of being discharged with an opioid prescription for non-Hispanic Black (NHB) compared to non-Hispanic White (NHW) patients. There was no statistically significant difference in odds of prescribing opioids by both White physicians and non-White physicians. In patients with the most severe fractures, requiring sedation for reduction, NHB patients had lower odds of receiving an opioid prescription (OR 0.80; 95% CI: 0.65–0.98).

**Conclusion:**

Within our institution, NHB patients received fewer opioid prescriptions at discharge for UE fractures. There is no statistically significant association between NHB race and odds of receiving an opioid prescription. In patients sedated for fracture reductions, NHB patients had lower odds of receiving an opioid prescription and non-White physicians had lower odds of prescribing opioids to NHB patients compared to NHW patients.

## 1 Introduction

Pediatric pain management in the emergency department (ED) has multiple challenges. Pain is often challenging for physicians to assess and difficult for patients to describe. Pediatric pain is often poorly recognized, poorly treated, and poorly documented. Multiple factors make pain management difficult, including the lack of pediatric pain management guidelines, decreased access to pediatric emergency care, and insufficient training in the appropriate treatment of acute pediatric pain [1].

Racial and ethnic disparities in health care have been well documented in the medical literature and exist across all aspects of healthcare [2]. Racial and ethnic disparities affect access to affordable and consistent care. These disparities are known to impact stress and lower overall life expectancy [3,4]. Additionally, they affect ED wait times, hospital admission rates, closed head trauma management, pain management and pain medication administration [[5], [6], [7], [8], [9], [10]].

Racial minorities, namely non-Hispanic Black (NHB) children, receive opioid analgesics less often than non-Hispanic White (NHW) children when they present to the ED [11,12]. Tomaszewski et al. evaluated opioid-prescribing patterns across multiple demographic factors and pain diagnoses. Bone injuries had the highest rate of opioid prescribing with 41.3% of these visits receiving an opioid prescription at discharge. Overall, White patients were prescribed opioids at a higher percentage than non-White patients [13]. To our knowledge, there have been few studies that have investigated the connection between opioid-prescribing practice and the race of not only the patient, but also of the prescribing physician.

The objective of this study was to examine the effect of self-identified race on opioid prescription practices and prescribing rates, relative to both the race of the patient and the prescriber in the pediatric emergency department (PED). We hypothesized the following: 1) NHB and Hispanic children with upper extremity fractures will receive fewer prescribed opioids (i.e. lower odds of prescription) than NHW children, 2) both White and non-White physicians prescribe fewer opioids to non-White children as compared to White children, 3) NHB and Hispanic children with the most severe fractures, requiring reduction under sedation, will also receive fewer prescribed opioids than NHW children.

## 2 Methods

### 2.1 Study design and setting

A cross-sectional, retrospective electronic medical record (EMR) review and physician survey were used to study opioid prescribing practices for upper extremity fractures, and to assess if the association between prescribing practices and patient race is modified by the self-identified provider race.

This study was submitted to and approved by our institutional review board (IRB). STUDY00000261.

### 2.2 Population

Our study was performed at a large, multi-campus tertiary care children's healthcare system in the southeastern region of the United States. The hospital system includes a level I trauma center serving urban/suburban/mixed population, a level II trauma center serving a more suburban mixed payer status population, and a community hospital serving an urban population. The three emergency departments at these campuses see roughly 240,000 combined patient visits annually.

All physicians who actively practiced in the PED at these three campuses during the study time period were surveyed. Study participation was voluntary and only patients seen by physicians who agreed to participate were included. Trainees (both residents and fellows) were excluded. Physicians without an active Drug Enforcement Administration (DEA) license were excluded.

### 2.3 Study protocol

Data were obtained via electronic medical record (EMR) chart review. The race of the patient was based on demographic information documented in the patient's medical chart, which is self-reported by the patient and his/her family. Other demographic data including age, gender, and payor status were also obtained.

Our EMR was used to identify patients ages 2–15 years presenting to the PED from January 2017 to July 2020 with the primary diagnoses of reduction of a left or right upper extremity fracture, radius fracture, ulnar fracture, supracondylar fracture, left or right forearm fracture, and left or right forearm injury, as identified by an ICD-10 code (Q71.91, Q71.92, S62.102A, S62.102B, S62.101A, S62.101B, S52.561A, S52.561B, S52.561C, S52.381A, S52.531A, S52.571A, S52.562A, S52.562B, S52.562C, S52.382A, S52.532A, S52.572A, S52.601A, S52.601B, S52.601C, S52.621A, S52.691A, S52.691B, S52.691C, S52.602A, S52.602B, S52.602C, S52.622A, S52.692A, S52.692B, S52.692C, S42.411A, S42.411B, S42.412A, S42.412B, S42.413A, S42.413B). All genders and all languages were included in this study. Patients less than 2 years were excluded as they often necessitate abuse evaluations by our trauma surgeons. In congruence with Adult Trauma Life Support (ATLS) guidelines, patients greater than 15 years with bony trauma are often seen at adult facilities and were excluded.

Physician self-reported race was obtained via an online survey, utilizing REDCapã. The survey was emailed to all physicians in the PEDs at all three campuses of the healthcare system. After the survey was generated, it was emailed to all eligible physicians via a secure link. Two separate reminder emails were sent two weeks apart. After the 2nd reminder email, it was assumed that all study participants had been included and the survey was closed. The survey consisted of five questions. Most importantly, one question asked the physician to “self-identify” his or her race. The physician was also asked for his or her name and demographic information such as number of years in practice and practice location. This information was used only to determine if the physician self-identified as White or non-White. Once charts were obtained, the physician's name was removed and not included in the data set to preserve anonymity. Only the self-identified race was reported.

### 2.4 Outcomes

Primary outcome measures included opioid medications prescribed at the time of discharge such as hydrocodone, hydrocodone derivatives, oxycodone, and oxycodone derivatives. Associations with the race of the physician and the race of the patient were assessed as the primary exposures. Opioids administered during the PED visit, whether given orally, intravenously, or intranasally, were not assessed.

### 2.5 Data analysis

Descriptive statistics were reported as medians and interquartile range (25th–75th) for continuous variables and counts with percentages for categorical variables. For bivariate analyses, continuous data were compared using Wilcoxon rank-sum tests, and categorical data were compared using Chi-squared tests of independence or Fisher's exact tests, if expected cell counts were <5. Standardized mean differences (effect sizes) were calculated for comparisons of opioid prescribed vs. not prescribed visits [14]. Logistic regression models were run to assess the association of patient race with the odds of having an opioid prescription at ED discharge. Since physicians saw multiple patients, all models accounted for the nesting of patients within physicians. Adjusted models, controlling for patient age, gender, fracture type, severity (sedation), and insurance status, are reported. An interaction term for physician race and patient race is included to evaluate if physician race (White vs. non-White) modified the association between patient race (NHW, NHB, Hispanic, other) and the odds of opioid prescription at ED discharge. If the interaction term was not significant (p < 0.05), the interaction term was dropped from the models and overall estimates for patient race (regardless of physician race) were calculated. Odds ratios with 95% confidence intervals and p-values were reported. Statistical significance was assessed at the 0.05 level. All statistical analysis was performed using SAS v9.4 (Cary, NC).

## 3 Results

### 3.1 Physician characteristics

There were a total of 140 eligible physicians. Ninety-four of these physicians consented to having their opioid prescribing practices reviewed. Of the 94 consenting physicians, 40 primarily work at the level II trauma center only and 54 work at the level I trauma center and community hospital. Forty-four physicians self-identified as White and 50 self-identified as non-White.

### 3.2 Patient characteristics

A total of 9225 unique patient visits were included initially in the study population. After excluding patient visits without physician race (if physician did not consent) and visits seen by fellows, there were 8156 unique patient visits left. One additional patient was excluded after being admitted, leaving n = 8155 patients for analysis. The median age was 8 (IQR: 5, 11) years old. Forty-six percent of patients ranged from 6 to 10 years of age. We found that 43% of patients were NHW, 33% of patients were NHB, 16% were Hispanic, and 6% were Other. One percent declined to answer. Most patients identified as male. Patients with Medicaid and commercial insurance were nearly equal, at 43% and 47%, respectively. Unknown insurance status accounted for 10% of patients (Table 1).

**Table 1:** Study demographics.

Physician Characteristics, N = 94	N (%) or Mean ± SD or Median (25th–75th)
Campus	
Scottish Rite	40 (43%)
Egleston and Hughes Spalding	54 (57%)
Race	
White	44 (47%)
Non-White	50 (53%)
**Patient Characteristics, N** = **8155 patients**	
Patient age, years, mean ± SD	8.1 ± 3.5
Patient age, years (group)	
2–5	2142 (26%)
6–10	3718 (46%)
11–15	2295 (28%)
Patient Gender	
Female	3062 (38%)
Male	5093 (62%)
Patient Race	
NH White	3514 (43%)
NH Black	2664 (33%)
Hispanic	1341 (16%)
Other	523 (6%)
Declined	113 (1%)
Patient Insurance	
Medicaid	3539 (43%)
Commercial	3795 (47%)
Unknown	821 (10%)
Required Procedural Sedation	2877 (35%)
Fracture (Bone involved)	
Humerus	1629 (20%)
Radius	5897 (72%)
Ulna	545 (7%)
Other	84 (1%)
**Patient Clinical Info, N** = **8155 patients**	
ED visits with opioids prescribed	2754 (34%)
Total opioid doses (n = 2754), median (25th – 75th)	10 (8.9, 12)

Note: 94 unique physicians for the 8155 ED patients (ranging from 3 to 451 admissions per physician, with a median of 67 admissions per physician in this dataset).

SD: standard deviation.

Radius fractures were the most common upper extremity fracture. Thirty-five percent of patients required procedural sedation for fracture reduction during their ED visits. A total of 2754 of patients received an opioid prescription on discharge. The median number of doses prescribed was 10. This is shown in Table 1.

Bivariate analysis of patients with (N = 2754) and without (N = 5401) opioid prescriptions are shown in Table 2. Of the patients who did receive an opioid prescription, the majority were male, 6–10 years of age, had commercial insurance, were NHW, and had a radius fracture. Most patients who received an opioid prescription were also seen by White physicians. In comparison, of the patients who did not receive an opioid prescription the majority were also male, 6–10 years of age, had Medicaid, were NHW, and had a radius fracture. Sixty percent of patients who did not receive an opioid prescription were seen by White physicians.

**Table 2:** Bivariate analysis of ED visits with and without opioid prescriptions.

	No Opioid prescribed, N = 5401	Opioid prescribed, N = 2754	P-value	ES
Patient age, years, mean ± SD	7.9 ± 3.5	8.6 ± 3.3	<0.001	0.187
Patient age, years (group)			<0.001	0.187
2-5	1563 (29%)	579 (21%)		
6-10	2398 (44%)	1320 (48%)		
11-15	1440 (27%)	855 (31%)		
Patient Gender			0.001	0.077
Female	2096 (39%)	966 (35%)		
Male	3305 (61%)	1788 (65%)		
Patient Race			<0.001	0.222
NH Black	1926 (36%)	738 (27%)		
NH White	2138 (40%)	1376 (50%)		
Hispanic	909 (17%)	432 (16%)		
Other	352 (6.5%)	171 (6%)		
Declined	76 (1%)	37 (1%)		
Patient Insurance			<0.001	0.212
Medicaid	2522 (47%)	1017 (37%)		
Commercial	2344 (43%)	1451 (53%)		
Unknown	535 (10%)	286 (10%)		
Sedation- Yes	1250 (23%)	1627 (59%)	<0.001	0.784
Fracture Type				
Humerus	1187 (22%)	442 (16%)	<0.001	0.152
Radius	3772 (70%)	2125 (77%)	<0.001	0.166
Ulna	369 (7%)	176 (6%)	0.450	0.018
Other	73 (1%)	11 (0.4%)	<0.001	0.102
Race of Physician			<0.001	0.105
White	3215 (60%)	1497 (54%)		
Non-white	2186 (40.5%)	1257 (46%)		

P-value: Wilcoxon rank-sum tests or Fisher's exact tests.

SD: standard deviation.

ES: effect size; Cohen's D criteria can be used (0.2-small, 0.5-medium, 0.8-large) for the reported ES. ***Sample sizes of this magnitude reduce the usefulness of p-values, as small quantitative differences often flag as statistically significant. The ES represents a standardized difference, irrespective of p-values, in determining practical (or clinical) significance of a result.

Column percentages are shown above.

Logistic regression models evaluated the odds of opioid prescription across patient and physician races, and results are shown in Table 3a, Table 3ba and 3b. Logistic regression models evaluating the odds of receiving an opioid prescription across patient race, without consideration of physician race (Table 3a), did not show any statistically significant difference. In Table 3b, the odds of opioid prescription by Non-White and White physicians for NHB patients did not show any statistically significant difference.

**Table 3a:** Logistic Regression Model Comparing Differences in Odds of Opioid Prescription across Patient Race (interaction term for physician race omitted).

Predictor	Odds Ratio	95% CI	P-value
**Patient Race**			
NH Black (vs. NH White)	0.90	0.78–1.03	0.135
Hispanic (vs. NH White)	1.00	0.85–1.18	0.984
Other (vs. NH White)	0.87	0.70–1.08	0.192

Comparisons using cluster (physician) adjusted logistic regression, controlling for patient age, gender, fracture type, severity (sedation), and insurance.

N = 8042 ED visits modeled above (excluded where patient race was declined).

**Table 3b:** Logistic Regression Model Comparing Differences in Odds of Opioid Prescription across Patient Race (including physician race interaction term).

	Predictors	Odds Ratio	95% CI	P-value
**Physician Race**	**Patient Race**			
Non-White	NH Black (vs. NH White)	0.89	0.73–1.09	0.268
	Hispanic (vs. NH White)	1.07	0.83–1.39	0.589
	Other (vs. NH White)	0.87	0.62–1.23	0.439
White	NH Black (vs. NH White)	0.92	0.77–1.10	0.334
	Hispanic (vs. NH White)	0.95	0.78–1.16	0.599
	Other (vs. NH White)	0.87	0.66–1.14	0.299

Comparisons using cluster (physician) adjusted logistic regression, controlling for patient age, gender, fracture type, severity (sedation), and insurance.

Patient race and physician race interaction term was tested: Interaction term p-value: 0.837, N = 8042 ED visits modeled above (excluded where patient race was declined).

### 3.3 Pediatric procedural sedation (PPS) sub-analysis

Patients with most severe upper extremity fractures often require bedside reductions with PPS. In order to account for this injury severity, a subset analysis was performed for PPS patients (Table 4a, Table 4ba and 4b). Logistic regression models were completed on the subset of PPS patients to compare odds of opioids being prescribed across patient race without the physician interaction. The odds of an opioid prescription were lower for NHB patients compared to NHW patients (Table 4a). Additionally, logistic regression models were completed to compare the odds of opioids being prescribed across patient race (including physician race interaction) in patients requiring PPS (Table 4b). In visits seen by Non-White physicians, odds of prescribing opioids to NHB patients were lower compared to NHW patients. For visits managed by White physicians, there was no statistically significant difference.

**Table 4a:** Logistic Regression Model Comparing Differences in Odds of Opioid Prescription across Patient Race (interaction term for physician race omitted) in Sedated Patients.

Predictor	Odds Ratio	95% CI	P-value
			
**Patient Race**			
NH Black (vs. NH White)	0.80	0.65–0.98	0.033
Hispanic (vs. NH White)	1.03	0.81–1.32	0.804
Other (vs. NH White)	0.92	0.68–1.25	0.591

Comparisons using cluster (physician) adjusted logistic regression, controlling for patient age, gender, fracture type, and insurance.

N = 2834 ED visits modeled above (excluded where patient race was declined).

**Table 4b:** Logistic Regression Model Comparing Differences in Odds of Opioid Prescription across Patient Race (including physician race interaction term) in Sedated Patients.

	Predictors	Odds Ratio	95% CI	P-value
				
**Physician Race**	**Patient Race**			
Non-White	NH Black (vs. NH White)	0.70	0.52–0.94	0.018
	Hispanic (vs. NH White)	1.09	0.74–1.60	0.671
	Other (vs. NH White)	0.94	0.57–1.53	0.788
White	NH Black (vs. NH White)	0.89	0.68–1.16	0.389
	Hispanic (vs. NH White)	1.01	0.74–1.36	0.976
	Other (vs. NH White)	0.91	0.62–1.34	0.627

Comparisons using cluster (physician) adjusted logistic regression, controlling for patient age, gender, fracture type, and insurance.

Patient race and physician race interaction term was tested: Interaction term p-value: 0.571, N = 2834 ED visits modeled above (excluded where patient race was declined).

## 4 Discussion

The results of this retrospective cross-sectional study, analyzing the disparities associated with opioid prescribing practices in 8155 PED patient visits for upper extremity long bone fractures did not reveal any statistically significant difference in the odds of NHB patients receiving fewer opioid prescriptions compared to NHW patients. In patients sedated for fracture reductions (presumably those with the more serious fracture), NHB patients had lower odds of receiving an opioid prescription from non-White physicians. White physicians had similar opioid prescription practices across all races.

Numerous studies have assessed the effects of race and ethnicity on analgesia administration, namely with patients with long bone fractures [2,[15], [16], [17], [18], [19], [20], [21], [22], [23]]. Most studies assessing the disparities associated with oligoanalgesia for long bone fractures have been performed in adult populations [2,15,16,18,19,22,23]. Few studies have been conducted in pediatric populations [17,20] and even fewer assessed both pediatric and adult patients [21]. The results of these studies have varied results and are poorly replicated [2,[15], [16], [17], [18], [19],[21], [22], [23]]. Goyal et al. reported minority children were more likely to receive any analgesic and report a two-point pain reduction however, were less likely to receive opioids and achieve optimal pain reduction in children with long bone fractures. These findings held across all racial demographics, even after adjusting for confounders [20]. Although Tamayo-Sarver et al. demonstrated disparities in opioids prescribed for patients presenting with migraines and back pain, the results did not reveal a statistically significant association between race and opioids prescribed for long bone fractures [24].

It has been documented that non-White patients are more likely to report severe pain to healthcare providers [20,25]. Quazi et al. and Tamayo-Sarver et al., suggest racial disparities exist for subjective pain such as headaches and back pain but not for objective and severe pain such as pain associated with long bone fractures [18,24]. Even when the pain is reported as severe, non-White patients are less likely to receive any analgesia or opioids [20,25]. The conclusions of Todd et al., suggest alternative reasons for non-White patients disparately receiving any analgesia or opioids since their study did not show any significant difference between patient or physician pain assessments [26].

In our study, we demonstrate an association between race and the receipt of a prescription opioid for long bone fractures. Overall, NHB patients received fewer opioid prescriptions at discharge for long bone fractures. However, when comparing the differences in the odds of receiving an opioid prescription between NHW patients and patients from other racial backgrounds, there was no statistically significant difference found. Assessments of the association between physician race and these findings again did not reveal a statistically significant difference, except when examining the subgroup of patients requiring PPS. NHB patients had lower odds of receiving an opioid prescription than NHW patients and, NHB patients had lower odds of receiving opioid prescriptions than NHW patients if seen by non-White physicians.

In comparison to displaced fractures or fractures with vascular compromise, nondisplaced fractures such as buckle (torus) fractures usually do not require reduction and can be managed with splinting or temporary casting [27,28]. In an effort to control for pain severity, a sub-analysis was performed on patients that underwent procedural sedation for fracture reduction in the PED. These results revealed disparities in the prescribing of opioids to NHB patients. When assessing for an association between physician race, non-White physicians had lower odds of prescribing opioids to NHB children than NHW children. This suggests that disparate care exists for pain control in children with severe long bone fractures, but this was only shown for non-White physician prescribers. Our institution does not have standardized pain prescription order sets and discharge instructions. It is possible that standardizing institutional pain management instructions and prescription guidelines would help to decrease some of these discrepancies [[29], [30], [31], [32], [33], [34]].

The results of our study add to the current literature. There have been few studies assessing the effect of physician race and characteristics on prescribing patterns [[35], [36], [37]], and to our knowledge, no studies evaluating association between physician race and patient race in relation to prescription of opioids for long bone fractures in pediatric patients. A recent study by Greenwood et al., revealed that physician-patient race concordance is associated with decreased mortality for Black infants [38]. Cheng et al. [39] and Rich [40] both propose the benefits of racial concordance on enhanced pain communication and patient satisfaction, respectively. In our study, we cannot say with certainty that physician-patient race concordance or non-concordance has any effect on the prescribing of opioids for long bone fractures. However, in our study there is evidence in disparate care for patients requiring PPS and reduction for the management of their fractures.

### 4.1 Limitations of study

There are several limitations to this study. This study is a single healthcare system study and therefore, the results may not be generalizable to national prescribing practices. However, our institution has one of the largest emergency medicine programs in the country and, in terms of pediatric emergency medicine faculty, it is one of the most racially diverse [[41], [42], [43]]. Additionally, this is a retrospective chart review. Therefore, there is the potential for misclassification of encounters based on diagnoses and diagnosis codes. Physicians were surveyed and only those physicians agreed to having their charts reviewed were included in the study. To keep individual physicians anonymous, we divided physicians into non-White and White racial categories instead of categories in accordance with the patient categories. We elicited the number of years physicians practiced and whether they were trained in emergency medicine however, we did not utilize this information in our analysis. We did not have the information for pain scores or pain reassessments available for analysis. Lastly, we only assessed opioids prescribed on discharge. We did not assess any analgesics provided during ED visits and did not assess over-the-counter medication recommendations provided on discharge.

## 5 Conclusion

Our study shows that there are racial and ethnic disparities in opioid prescribing practices for upper extremity injuries in the PED. NHB children with the most severe fractures, requiring PPS for reduction in the PED, had lower odds of receiving opioid pain medications by a non-White physician at the time of discharge. Further research into prescribing practices, the role of individual biases, and patient outcomes on a national level is needed in order to improve care for minoritized children.

**Article summary**

1. Why is this topic important? There are well documented disparities regarding pain management in the emergency setting. This study assesses if non-Hispanic Black and Hispanic patients received fewer opioids than White patients and if the self-identified race of the provider played a role.
2. What does this study attempt to show? If racial concordance and discordance is associated with disparate care, regarding opioid prescribing practices for pediatric patients with long bone fractures.
3. What are the key findings? In patients sedated for fracture reductions, non-Hispanic Black patients had lower odds of receiving an opioid prescription and non-White physicians had lower odds of prescribing opioids to non-Hispanic Black patients compared to non-Hispanic White patients.
4. How is patient care impacted? Patient care is impacted due to the under management of pain and disparate care in non-Hispanic Black patients with severe fractures requiring reduction.

## Author contribution statement

Joseph Langham, MD; Sherita L. Holmes, MD, FAAP; Carmen D. Sulton, MD, FAAP, FACEP: Conceived and designed the experiments; Performed the experiments; Analyzed and interpreted the data; Wrote the paper.

Janet Figueroa: Performed the experiments; Analyzed and interpreted the data; Contributed reagents, materials, analysis tools or data; Wrote the paper.

Srikant Iyer, MD, MPH; Sarah Lazarus, DO: Conceived and designed the experiments; Analyzed and interpreted the data; Wrote the paper.

Scott Gillespie: Conceived and designed the experiments; Performed the experiments; Analyzed and interpreted the data; Contributed reagents, materials, analysis tools or data; Wrote the paper.

## Funding statement

This research did not receive any specific grant from funding agencies in the public, commercial, or not-for-profit sectors.

## Data availability statement

The data that has been used is confidential.

## Declaration of interest's statement

The authors declare that they have no known competing financial interests or personal relationships that could have appeared to influence the work reported in this paper.
